# p16 gene transfer increases cell killing with abnormal nucleation after ionising radiation in glioma cells

**DOI:** 10.1038/sj.bjc.6601299

**Published:** 2003-10-28

**Authors:** S Hama, S Matsuura, H Tauchi, F Yamasaki, Y Kajiwara, K Arita, H Yoshioka, Y Heike, K Mandai, K Kurisu

**Affiliations:** 1 Department of Neurosurgery, Hiroshima University School of Medicine, Kasumi 1-2-3, Minami-ku, Hiroshima 734-8551, Japan; 2 Department of Radiation Biology, Research Institute for Radiation Biology and Medicine, Hiroshima University, Kasumi 1-2-3, Minami-ku, Hiroshima 734-8553, Japan; 3 Division of Clinical Research, National Shikoku Cancer Center Hospital, Horinouchi 13, Matsuyama, Ehime 790-0007, Japan; 4 Division of Pathology, National Shikoku Cancer Center Hospital, Horinouchi 13, Matsuyama, Ehime 790-0007, Japan

**Keywords:** p16, ionising radiation, abnormal nucleation, apoptosis, human glioma cell line

## Abstract

It is well established that cells synchronised at the G1–S phase are highly radiosensitive. In this study, p16-null human glioma cell lines were induced into G1 cell cycle arrest by adenovirus-mediated p16 gene transfer, and examined for radiation-induced cell killing. Clonogenic analysis and trypan blue extraction test showed that the p16 gene transfer enhanced radiation-induced cell killing in p16-null glioma cell lines. TUNEL assays and pulse-field gel electrophoresis confirmed that the radiation-induced cell killing of p16-transfected cells could be caused by a nonapoptotic mechanism. Gimsa staining demonstrated that irradiation alone or Ax-mock infection plus irradiation results in a slight increase in the frequency of cells with abnormal nucleus, compared to unirradiated uninfected or Ax-mock infected cells. However, Ax-hp16 or Ax-hp21 infection alone modestly increased the frequency of cells with abnormal nucleus (especially bi- and multinucleation), and 4-Gy irradiation of Ax-hp16 or Ax-hp21 infected cells substantially enhanced this frequency. These results suggest that there exists some unknown interaction between radiation and p16 in cytoplasm/membranes, which decreases cytokinesis and promotes abnormal nucleation. Thus, p16 expression prevented radiation-induced apoptosis by promoting abnormal nucleation, thereby leading to another mode of cell death.

Human malignant gliomas diffusely invade surrounding normal brain tissue. Surgical removal of the entire tumour is difficult, and radiation therapy is often administered as an adjuvant in the treatment of incompletely resected tumours. However, the clinical effectiveness of irradiation has been limited. In almost all cases, tumours are refractory to current treatments, and the patient dies from brain herniation due to unrestrained growth of the tumour (Giangaspero and Burger, 1983). The median survival time of malignant glioma patients is less than 2 years, despite extensive and multidisciplinary treatment.

Glioma cells have been reported to display abnormality in one or more genes responsible for regulating cell-cycle progression. Such genes include the well-characterised tumour suppressor genes p53, pRb, cyclin D, p21 and p16 (Gomez-Manzano *et al*, 1997; Costanzi-Strauss *et al*, 1998; Buschges *et al*, 1999). p53 inhibits cell-cycle progression in response to various cellular stresses by direct transactivation of p21, an important mediator of cell proliferation, differentiation and senescence (el-Deiry *et al*, 1993; Xiong *et al*, 1993; Noda *et al*, 1994). p21 inhibits the ability of cyclin-dependent kinases (CDKs) to phosphorylate pRb, thereby inhibiting cell entry into the S phase. Similarly, p16 inhibits the ability of CDK4 and CDK6 to regulate the phosphorylation status of pRb, thereby also controlling progression into the S phase (Nobori *et al*, 1994; Lukas *et al*, 1995). Recently, several authors have shown that 4–52% of clinical samples of malignant glioma and 68–87% of glioma cell lines have a homozygous deletion of the p16 locus (He *et al*, 1994; Nobori *et al*, 1994; Schmidt *et al*, 1994; Ueki *et al*, 1996; Hama *et al*, 1997). Therefore, restoration of normal function of both p53 and p16 by gene therapy is an attractive strategy for glioma treatment.

Adenovirus-mediated gene transfer of wild-type p16 following irradiation has previously been shown (by clonogenic survival assay) to enhance radiosensitivity in lung non-small-cell cancer cells (Kawabe *et al*, 2000). The results of clonogenic survival assays clearly indicate that cells synchronised at the late S and early G1 phase are the most radioresistant, whereas cells synchronized at the M phase or at the late G1 to early S phase are the most radiosensitive (Terasima and Tolmach, 1963; Okada, 1970). The regulatory cascade comprising pRb, cyclin D1, CDK4/CDK6 and p16 plays a central role in cell-cycle control, and may govern radiosensitivity as well.

We have previously demonstrated that p16 gene transfer into human glioma cells using an adenovirus vector constructed with the COS-TPC method completely inhibits proliferation of the cells via G1 arrest for over 5 days (Hama *et al*, 1998). This adenovirus vector has a high transfection efficiency and provides stable, long-term gene expression. The purpose of this study was to determine whether adenovirus-mediated p16 gene transfer is a suitable gene replacement therapy for human glioma.

## MATERIALS AND METHODS

### Cells

U251MG cells (human glioma; p16, homozygous deletion; p21, undetectable protein expression; p53, mutated at codon 273 [CGT/CAT Arg/His]) and D54MG cells (human glioma; p16, undetectable protein expression; p53, wild type) were used in this study (Fueyo *et al*, 1996; Gomez-Manzano *et al*, 1996; Hama *et al*, 1998; Kawada *et al*, 1998). U251MG cells were obtained from the Japanese Cancer Research Resources Bank (Tokyo, Japan). D54MG cells were provided by Dr DT Curiel (Miller *et al*, 1998).

### Adenovirus vectors

The p16- or p21-expressing adenovirus vectors used here were also used in previous studies of ours (Hama *et al*, 1998; Yamasaki *et al*, 2003). In brief, replication-defective adenovirus vectors were based on the human adenovirus type 5(Ad5) serotype. The full-length human p16 cDNA was a gift from Dr T Nobori, and the full-length p21 cDNA was purchased from the Meiji Institute of Health (Nobori *et al*, 1994; Noda *et al*, 1994). Recombinant adenovirus vectors containing the full-length human p16 gene (Ax-hp16) or p21 gene (Ax-hp21) were generated using the COS-TPC method, as described elsewhere (Niwa *et al*, 1991; Miyake *et al*, 1996). Exogenic p16 or p21 gene expression after Ax-hp16 or Ax-hp21 infection has been achieved in previous studies (Hama *et al*, 1998; Yamasaki *et al*, 2003). Control vectors consisted of recombinant adenoviruses containing an expression cassette without any genome (mock adenovirus vector, Ax-mock). The optimal conditions of the virus infection of each cell line for adequate gene transfer were evaluated as described elsewhere (Hama *et al*, 1998).

For transient infection, the culture medium was replaced 2 h after infection with the adenovirus vector; for continuous infection, the medium was not changed until analysis. Adenovirus expression appeared to continue for 3–5 days after transient infection, and for over 5–7 days during continuous infection (Hama *et al*, 1998). In the present study, most gene transfer was performed using continuous infection.

### Irradiation and adenovirus infection

To obtain uniform and reproducible irradiation conditions, cells were trypsinised, counted, placed in the glass tube, and irradiated using a ^60^Co source at a dose rate of 0.65 Gy min^−1^, as described elsewhere (Tauchi *et al*, 2001). Known numbers of cells were then replated in culture dishes and returned to the incubator. One day after irradiation, U251MG cells were infected with adenovirus vector at 5 multiplicity of infection (MOI), and D54MG cells were infected at 10 MOI.

### Cell survival and cell viability analysis

Cell survival was assessed using clonogenic assays in monolayer culture, as described elsewhere (Tauchi *et al*, 2001). In brief, the cells were trypsinised, counted, placed in the glass tube and irradiated, as described above. Known numbers of cells were then replated in 100-mm culture dishes and returned to the incubator to allow macroscopic colony development. The surviving fractions following a given treatment were calculated based on survival of nonirradiated noninfected cells.

The viability of cells with or without irradiation (4 Gy) was analysed using trypan blue exclusion, as described elsewhere (Hama *et al*, 1998). Each experiment was repeated at least twice.

### Analysis of cell cycle by flow cytometry

Cell-cycle of U251MG cells and D54MG cells was assessed using flow cytometry, as described elsewhere (Hama *et al*, 1998). In short, these cells were plated in a 100-mm dish and infected with Ax-hp16 or Ax-mock after irradiation. The cells were collected by trypsinisation after 3 or 6 days of incubation, washed twice with PBS, fixed with 75% ethanol and maintained at 4°C for 48 h. After centrifugation at 1500 rpm for 10 min, the cells were washed with PBS and resuspended in 1 ml of lysis buffer (0.1% Triton-X 100, 0.1% RNaseA) at 4°C overnight to free their nuclei. Immediately before analysis, 1 ml of 50 *μ*g ml^−1^ propidium iodide (PI) solution (final concentration, 25 *μ*g ml^−1^) was added to the cell samples. Propidium iodide fluorescence of individual nuclei was measured using a FACscan (Becton Dickinson Co., Lincoln Park, NJ, USA). Data were analysed with the ModFit LT program (Becton Dickinson Co., Lincoln Park, NJ, USA). Each experiment was repeated at least twice.

### Detection of apoptosis

Radiation-induced apoptosis was assessed morphologically by TUNEL assay using the *In Situ* Apoptosis Detection Kit (Cat. MK500, Takara Bio Inc., Ootsu, Japan). Cells were irradiated with a dose of 4 Gy, and were infected with Ax-mock, Ax-hp16 or Ax-p21 as described above. These cells were cultured in chamber slides for further 2 or 6 days. Then, after fixation with 4% formaldehyde, they were stained according to the manufacturer's instructions, and photographs were taken under 160 × magnification, using a Nikon OPTIPHOT-2 fluorescence microscope. The apoptotic index (AI) was defined as the percentage of TUNEL-positive cells in a 200 × magnified field, and the AI values of the experimental groups were compared. Two different people blinded to the treatment counted the positive cells in three microscopic fields on one slide from each specimen; there were no significant differences between the counts they obtained. Each experiment was repeated at least twice.

### Pulse-field gel electrophoresis (PFGE)

Induction of cleavage of chromatin in U251MG cells was determined by PFGE, as described elsewhere (Hwang *et al*, 2000; Cipriani *et al*, 2001). In breif, U251MG cells were infected with Ax-hp16 or Ax-mock after irradiation and collected by trypsinisation after 5 days of incubation. Cell pellets containing 2 × 10^7^ U251MG cells/sample were resuspended in 1000 *μ*l of Ca^2+^–Mg^2+^-free PBS and 1000 *μ*l of 1% low-melt agarose, and solidified at 4°C for 30 min. Plugs were digested for 48 h at 55°C in 5 ml of digestion buffer containing 100 mM EDTA (pH 8.0), 0.2% sodium deoxycholate, 1% sodium lauryl sarcosine, and 1 mg ml^−1^ proteinase K. The plugs were then washed four times in washing buffer (20 mM Tris-HCl (pH 8) and 50 mM EDTA), loaded into a 1.2% agarose gel, and run in 0.5x TBE buffer for 22 h using the CHEF-DR II electrophoresis cell (Bio-Rad, Hercules, CA, USA). Running conditions were optimised to separate 50–1000-kbp DNA fragments, as follows: temperature, 14°C; switch time, 50–90 s; angle, 120°; voltage gradient, 6 V cm^−1^. The standard-size DNA ladder was included. After staining with 0.5 *μ*g ml^−1^ ethidium bromide, bands were visualised using a UV transilluminator (254–360 nm).

### Evaluation of nuclear morphology

Nuclear morphologic changes in U251MG or D54MG cells were evaluated using Giemsa staining, as described elsewhere (Bhattathiri *et al*, 1998a, 1998b; Bhattathiri, 2001). U251MG and D54MG cells were irradiated with a dose of 4 Gy, and were infected with Ax-mock, Ax-hp16 or Ax-p21 as described above. These cells were cultured for a further 6 days, collected by trypsinisation, spread on a clean glass slide, fixed in ethyl alcohol and stained with Giemsa stain. The frequency of micronucleated, nuclear budded, binucleated and multinucleated tumour cells was evaluated. The criteria for identification of these cells are described elsewhere (Bhattathiri *et al*, 1998a, 1998b; Bhattathiri, 2001). Briefly, micronucleated cells contain small round bodies of nuclear material within the cytoplasm, separate from the main nucleus (the cells indicated with one arrow in Figure 4D and E). Nuclear budded cells have buds adjoining the main nucleus; these could be micronuclei, if not for the fact that their separation from the main nucleus is indistinct (the cells indicated with two arrows in Figure 4D and E). Binucleated cells have two nuclei. Multinucleated cells have more than two nuclei (Figure 4C and the cells indicated with three arrows in Figure 4E). Around 500–1000 cells were evaluated from the samples collected on each occasion, and the results were expressed in comparison with 1000 culture cells with a single nucleus (ie, uninucleated cells). This experiment was repeated at least twice.

### Statistical analysis

Statistical analysis was performed by Student's *t*-test using Stat View 5.0 (Abacus Concepts, Inc). A *P*-value of <0.05 was considered to indicate statistical significance.

## RESULTS

### Effect of p16 gene transfer on radiation-induced cell killing

We first tested whether Ax-hp16 infection increased the radiosensitivity of human glioma cell lines *in vitro*. Clonogenic assays were performed on two glioma cell lines: U251MG (p16-null, p21-null and p53-mutant) and D54MG (p16-null and p53-wild type). These cell lines were infected with either Ax-hp16 or Ax-mock after irradiation. In preliminary experiments, we compared the effect on radiosensitivity between various time courses of irradiation and infection (transient infection *vs* continuous infection). The maximum effect was obtained when cells were continuously infected with Ax-hp16 after irradiation. We also assessed continuous adenovirus infection before irradiation; however, this procedure required collection of cells by trypsinisation after infection, transfer of the medium plus cells and adenovirus vectors to a glass tube, irradiation and replating of irradiated cells on the culture dishes without exchanging the medium (containing the adenovirus vector). This procedure was intricate and not reproducible. Therefore, we chose to irradiate cells prior to adenoviral infection. As continuous adenovirus infection is particularly likely to cause direct cytotoxicity, we assessed the cytotoxicity of the vector alone using plating efficiency as the end point. Cytotoxicity became evident at MOI values between 5 and 10 in U251MG cells, and between 7.5 and 15 in D54MG cells. Then, in further preliminary experiments, we used MOI values of 5 and 10 in U251MG cells and MOI values of 7.5, 10 and 15 in D54MG cells to assess radiosensitisation (data not shown). For U251MG, the most effective radiosensitisation was achieved using an MOI value of 5; for D54MG, this MOI value was 10.

**Figure 1:**
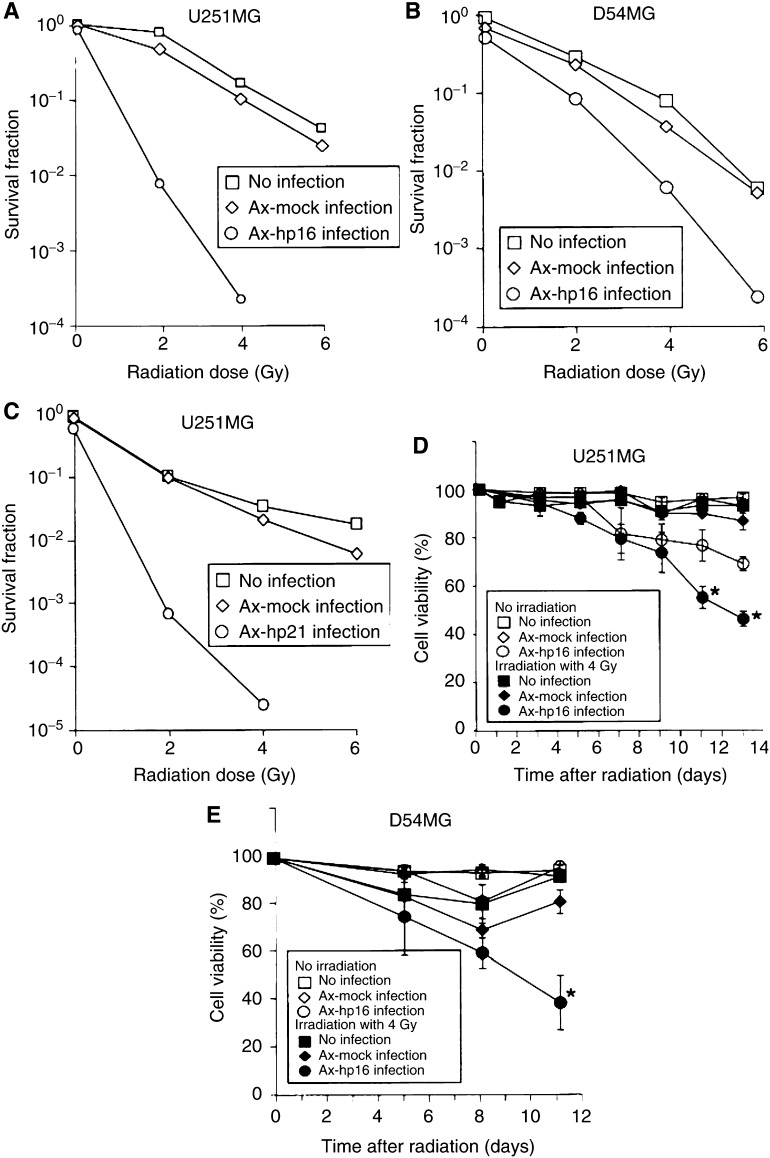
Effect of Ax-hp16 or Ax-hp21 infection on cell survival or cell viability in p16- or p21-null human glioma cell lines with or without irradiation. At 1 day after irradiation, U251MG cells (p16-null, p21-null, p53-mut) were infected with adenovirus vector at 5 MOI, and D54MG cells (p16-null, p53-wild) were infected at 10 MOI. (**A**) U251MG, Ax-mock or Ax-hp16 infection; (**B**) D54MG, Ax-mock or Ax-hp16 infection; and (**C**) U251MG, Ax-mock or Ax-hp21 infection show cell survival determined by clonogenic assays. (**D**) U251MG, Ax-mock or Ax-hp16 infection and (**E**) D54MG, Ax-mock or Ax-hp16 infection show cell viability determined using Trypan blue exclusion. The results of cell viability assays are shown as the mean number of cells at each point, with standard deviation of three wells. Statistical significance: ^*^P<0.05, compared with cells other than irradiated Ax-hp16-infected cells.

As shown in Figure 1A

, the survival of nonirradiated U251MG cells was slightly reduced by Ax-hp16 infection to 83%; at 2 Gy, it was markedly reduced from 77 to 0.69%; at 4 Gy, it was reduced from 15 to 0.019%. The percent survival of nonirradiated D54MG cells was slightly reduced by Ax-hp16 infection to 55%; at 2 Gy, it was markedly reduced from 31 to 9.1%; at 4 Gy, it was reduced from 8.5 to 0.67% (Figure 1B). Ax-mock infection alone did not produce marked sensitisation in either of the two cell lines in the range of 2–6 Gy. U251MG cells or D54MG cells infected with Ax-hp16 were significantly more radiosensitive than noninfected or Ax-mock-infected cells. This trend was similar to that of Ax-hp21-infected U251MG cells (Figure 1C).

Next, we examined the cell viability of the U251MG and D54MG cells using the trypan blue exclusion test (Figure 1D, E). Until 5 days after irradiation, there was no difference in viability between Ax-h-p16-infected cells and Ax-mock-infected or noninfected cells. In contrast, 7–8 days after irradiation, the viability of irradiated Ax-hp16-infected cells gradually decreased, compared with Ax-mock-infected, noninfected and nonirradiated cells. The viability of nonirradiated Ax-hp16-infected U251MG cells decreased gradually. The viability of irradiated Ax-hp16-infected U251MG cells decreased more significantly than that of nonirradiated Ax-hp16-infected cells (*P*=0.005 on day 11, and *P*=0.031 on day 13).

Thus, adenovirus-mediated p16 gene transfer enhanced the radiosensitivity of glioma cell lines that lack p16, but contain either wt-p53 or mut-p53.

### Effect of p16 gene transfer on cell cycle of U251MG cells after irradiation

**Figure 2:**
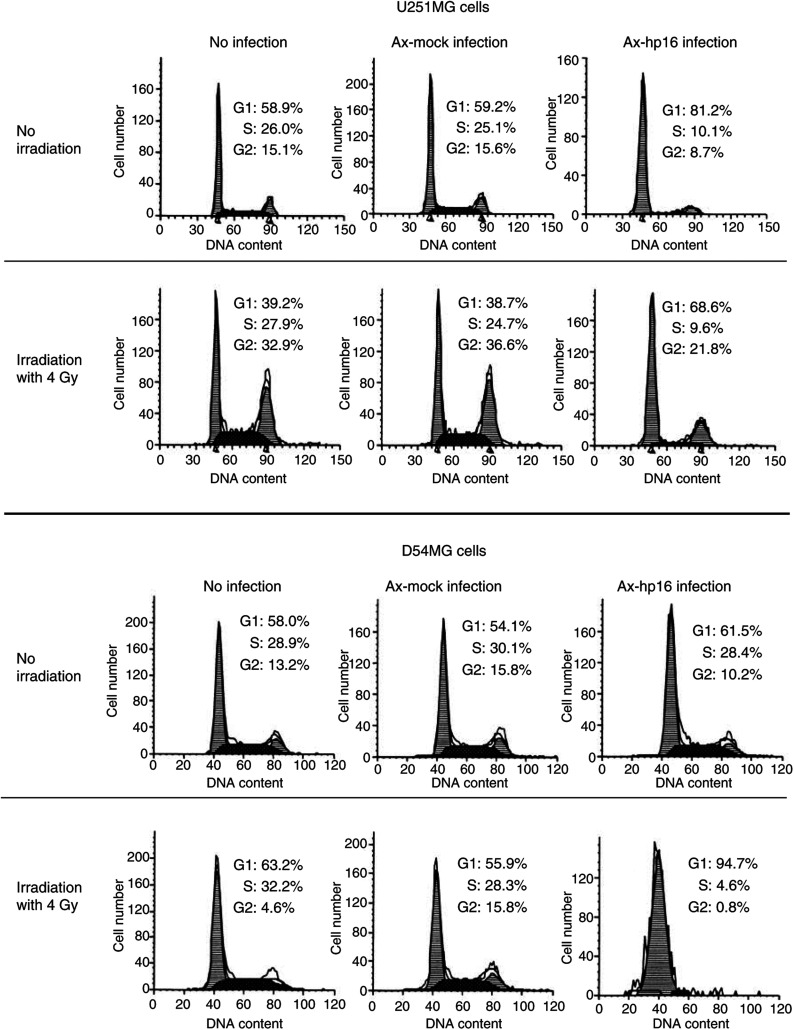
Effect of p16 transduction on cell-cycle of U251MG and D54MG cells. U251MG (upper) and D54MG cells (lower), nonirradiated or irradiated at a dose of 4 Gy, noninfected or infected with Ax-hp16 or Ax-mock, were cultured for 5 days, and the cell-cycle was analysed by flow cytometry. Data are presented as a histogram, with cell number (*Y*-axis) plotted against DNA content (*X*-axis). The first peak contains the cells with diploid DNA at G0/G1. The cells in the second peak with double PI-fluorescence intensity are tetraploid at G2/M. The area between the two peaks contains the cells in the S phase.

To determine whether Ax-hp16 infection induced G1 cell-cycle arrest in U251MG or D54MG cells, cell-cycle status was analysed on days 3 and 6 using flow cytometry. Figure 2

shows the histogram of cell-cycle status following irradiation and/or infection with Ax-mock or Ax-hp16 on day 6 (the data for day 3 were almost the same as that of day 6, and is not shown).

Nonirradiated Ax-hp16-infected U251MG and D54MG cells showed G1 arrest during the 6 days following infection, whereas the cell-cycle profile of Ax-mock-infected cells was the same as that of noninfected cells. In U251MG cells, after 4-Gy irradiation, G2/M accumulation occurred in Ax-mock-infected and noninfected cells. G2/M accumulation was markedly decreased in Ax-hp16-infected cells, compared with Ax-mock-infected and noninfected cells. In D54MG cells, after 4-Gy irradiation, G1 accumulation occurred in Ax-mock-infected and noninfected cells, and was significantly increased in Ax-hp16-infected cells. These results demonstrate that Ax-hp16 infection resulted in G1 arrest.

### Apoptotic analysis

**Figure 3:**
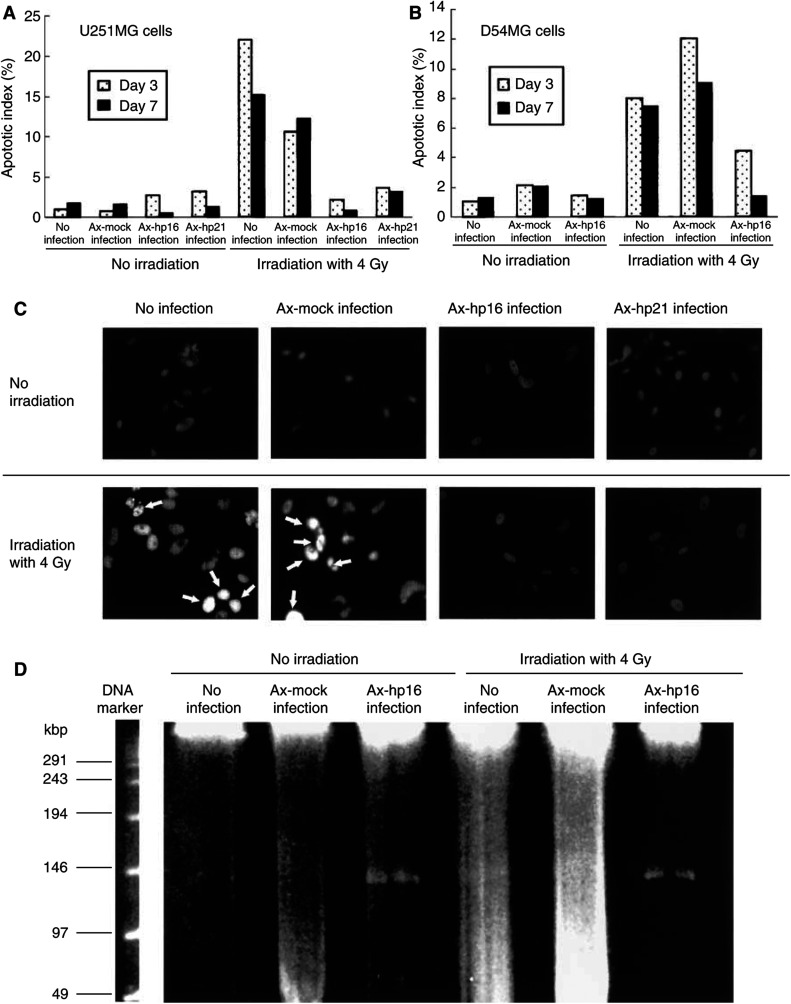
Apoptosis assessed by the TUNEL assay and PFGE. TUNEL assay was used for the morphological detection of apoptotic changes. U251MG or D54MG cells, nonirradiated or irradiated at a dose of 4 Gy, noninfected or infected with Ax-hp16, Ax-hp21 or Ax-mock 1 day after irradiation, were cultured for 2 or 6 days, and were stained according to the manufacturer's instructions after fixation with 4% formaldehyde. The AI was defined as the percentage of TUNEL-positive U251 (**A**) or D54MG cells (**B**). (**C**) TUNEL signals were detected by fluorescence microscopy in the nuclei of some irradiated uninfected or Ax-mock-infected U251MG cells (arrows). (**D**) The chromatin structure was analysed using PFGE. U251MG cells were treated as described in the TUNEL assay, and, 5 days after infection, cells were embedded in agarose gel and subjected to PFGE. The 50/300-kb fragments, which are generated during the early stage of apoptosis, were formed in irradiated noninfected and Ax-mock-infected U251MG cells. The sizes of the DNA fragments are indicated on the left.

To determine whether the death of Ax-hp16-infected U251MG cells after irradiation was due to apoptosis, we performed the TUNEL assay and measured the level of apoptosis on the basis of TUNEL positivity following various treatments 3 and 7 days after irradiation. Figure 3A

shows the percentage of TUNEL-positive cells among U251MG cells that were noninfected, Ax-mock-infected, Ax-hp16-infected or Ax-hp21-infected, with or without irradiation. Irradiated cells that were noninfected or Ax-mock-infected had a higher proportion of TUNEL-positive cells (uninfected cells, 22.0% on day 3 and 15.1% on day 7; Ax-mock infected cells, 10.6% on day 3 and 12.2% on day 7) than the corresponding unirradiated cells. Irradiated cells that were Ax-hp16- or Ax-hp21-infected had the same proportion of TUNEL-positive cells (Ax-hp16-infected cells, 2.1% on day 3 and 0.7% on day 7; Ax-hp21-infected cells, 3.6% on day 3 and 3.1% on day 7) as the corresponding unirradiated cells. D54MG showed a similar trend (irradiation alone, 8.0% on day 3 and 7.4% on day 7; Ax-mock infection plus irradiation, 12.0% on day 3 and 9.0% on day 7; Ax-hp16 infection plus irradiation, 4.4% on day 3 and 1.3% on day 7) (Figure 3B).

As shown in Figure 3C, irradiated noninfected or Ax-mock-infected U251MG cells were identified as scattered clusters of fluorescent staining, suggesting that the death of these cells was due to apoptosis. In contrast, Ax-hp16-infected U251MG cells did not emit fluorescent signals even after irradiation. p21 transfection caused a similar fluorescent staining in irradiated cells (Figure 3C). Therefore, it is unlikely that the cell death of irradiated Ax-hp16- or Ax-hp21-infected U251MG cells during the first 7 days after irradiation was due to apoptosis.

To confirm these interpretations, the chromatin structure was analysed using PFGE (Figure 3D). We investigated the cleavage of chromatin by examining the formation of 50/300-kb fragments, which are generated during the early stages of apoptosis and can be identified by PFGE (Khodarev *et al*, 1998; Hwang *et al*, 2000; Cipriani *et al*, 2001). The 50/300-kb fragments were formed in irradiated noninfected and Ax-mock-infected U251MG cells, but not in nonirradiated U251MG cells. Ax-hp16-infected U251MG cells did not form 50/300-kb fragments, with or without irradiation. These results support our hypothesis that cell death of Ax-hp16-infected U251MG cells following irradiation is not due to apoptosis.

### Nuclear morphology

**Figure 4:**
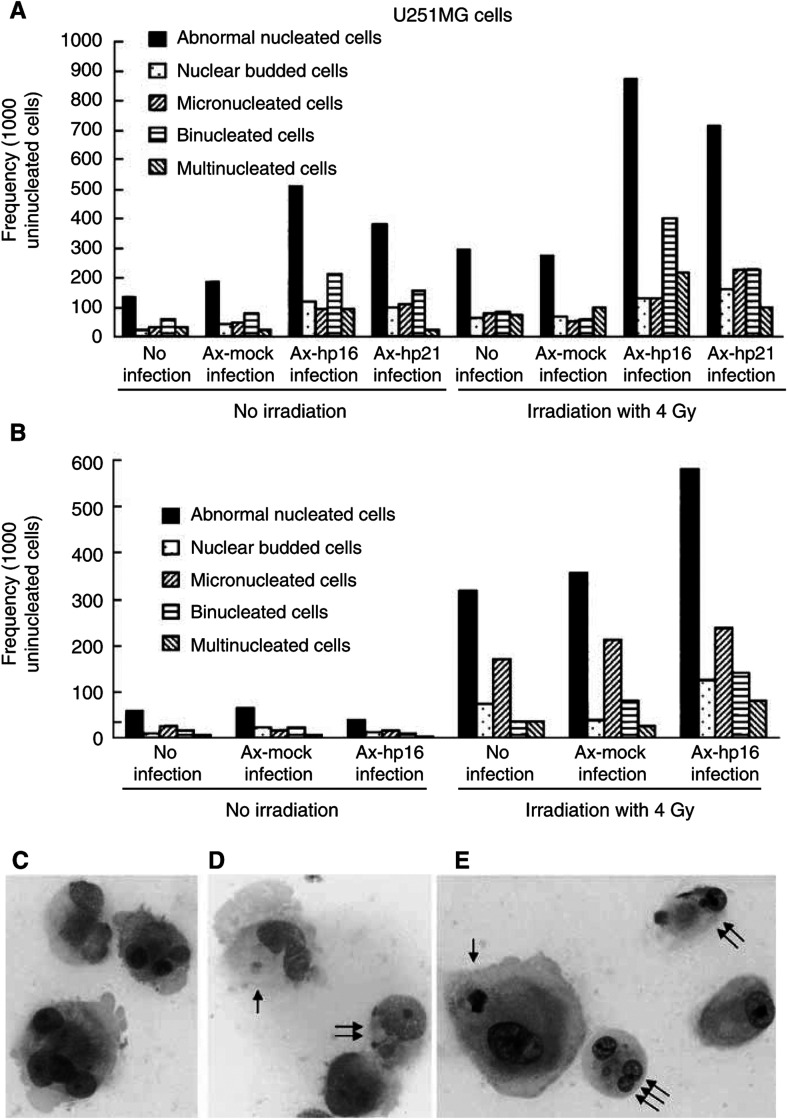
Effect of p16 or p21 transduction with or without irradiation on nuclear morphologic changes. U251MG or D54MG cells were treated as described in the TUNEL assay, and nuclear morphologic changes in these treated cells were evaluated using Giemsa staining 7 days after irradiation. The frequency of abnormal nuclei (including nuclear budding, micronucleation, binucleation and multinucleation) in U251MG (**A**) or D54MG cells (**B**) is expressed in comparison with 1000 culture cells with a single nucleus (ie, uninucleated cells). The figures show (**C**) multinucleation, (**D**) micronucleation (one arrow) and nuclear budding (double arrows) in irradiated Ax-hp16-infected U251MG cells, and (**E**) micronucleation (one arrow), nuclear budding (two arrows) and multinucleation (triple arrows) in irradiated Ax-hp16-infected D54MG cells.

To determine the nature of the radiation-induced cell death of the Ax-hp16- and Ax-hp21-infected cells, microscopic analysis using Giemsa staining was performed to measure the level of abnormal nucleus changes (budding and micro-, bi- and multinucleation). The morphological appearance of these nuclear changes is shown in Figure 4C, D and E

.

Figure 4A shows the frequency of cells with abnormal nucleus per 1000 uninucleated cells treated with noninfection, Ax-mock-, Ax-hp16- or Ax-hp21 infection, with or without irradiation, 7 days after irradiation. Irradiation alone and Ax-mock infection plus irradiation resulted in a slight increase in this frequency (uninfection, 64 budded, 75 micro-, 83 bi-, and 69 multinucleations; Ax-mock infection, 68 budded, 50 micro-, 54 bi-, and 100 multinucleations), compared to unirradiated uninfected or Ax-mock-infected cells. As expected, Ax-hp16 or Ax-hp21 infection alone modestly increased the frequency of cells with an abnormal nucleus, especially for bi- and multinucleation (Ax-hp16, 116 budded, 90 micro-, 207 bi-, and 90 multinucleations; Ax-hp21 infection, 97 budded, 108 micro-, 152 bi-, and 21 multinucleations), and 4-Gy irradiation of Ax-hp16- or Ax-hp21-infected cells substantially enhanced this frequency (Ax-hp16, 128 budded, 128 micro-, 398 bi-, and 214 multinucleations; Ax-hp21 infection, 158 budded, 227 micro-, 227 bi-, and 99 multinucleations), compared with the corresponding controls (Figure 4C, D). D54MG cells showed a similar trend (Figure 4B, E). These increases in the frequency of cells with an abnormal nucleus are consistent with increased abnormal nucleus formation resulting from the combined treatment of Ax-hp16 or Ax-hp21 infection plus irradiation.

## DISCUSSION

The inactivation of p16 function has been linked to the development of malignant gliomas, and recent investigations have provided an insight into the manner in which this inactivation may contribute to cell proliferation (Nobori *et al*, 1994; Fueyo *et al*, 1996; Hama *et al*, 1997, 1998; Costanzi-Strauss *et al*, 1998). Previous findings indicate that p16-dependent downmodulation of pRb levels in the presence of overexpressed wild-type p53 causes apoptosis only in tumour cells, whereas normal cells arrest in G1 (Sandig *et al*, 1997). Kawabe *et al* (2000) demonstrated, by clonogenic survival assay, that adenovirus-mediated p16 gene expression enhanced the radiosensitivity of non-small-cell lung cancer cells, depending on the endogenous wild-type p53 expression of the cell line. It is well established that radiation sensitivity varies with the population of the cell-cycle, and that cells at the M-phase and late G1 to the early S phase have great sensitivity (Terasima and Tolmach, 1963; Okada, 1970). Here, we assessed the ability of Ax-hp16 to enhance radiosensitivity in human glioma cell lines that lack p16, but have either wt-p53 or mut-p53.

The clonogenic survival assays indicated that the radiosensitivity of D54MG cells, which have wt-p53, was substantially increased by Ax-hp16 infection, as reported previously (Figure 1B). However, contrary to this previous report, the radiosensitivity of U251MG cells, which do not have functional p53, was also increased by Ax-hp16 infection (Figure 1A). As indicated by trypan blue exclusion test, U251MG and D54MG cells exhibited reduced viability after irradiation in combination with p16 gene replacement (Figure 1D–E). The results indicate that Ax-hp16 infection increased cell death in a p53-independent manner.

Based on these results, we doubt that the effect of Ax-hp16 infection on radiosensitivity was induced by the direct effect of p16 or by G1 arrest. Therefore, we used a clonogenic assay to assess the effects on the radiosensitivity of an adenovirus vector expressing another G1–S regulatory factor, p21. The results show that Ax-hp21 had the same effect as Ax-hp16 (Figure 1C). Moreover, we examined cell-cycle status using flow cytometry (Figure 2). Radiation alone produced G1 arrest in D54MG cells and G2-M arrest in U251MG cells. The observed difference in cell-cycle progression between the two cell lines after irradiation may be due to the difference in their p53 status, and is consistent with previous findings obtained using the same cell lines (Ostruszka and Shewach, 2000). However, Ax-hp16-infected U251MG and D54MG cells were arrested in the G1 phase during the 6 days following irradiation, suggesting that the induction of G1 arrest following irradiation increases the radiosensitivity of these cells.

Kawabe *et al* (2000) reported that p16 gene transfer enhanced apoptotic cell death for cells that have wt-p53, but not for p53-mutated or p53-deleted cells. The present findings indicate that p16 enhanced radiosensitivity via increased cell death, and that this enhancement did not depend on p53 status. We examined the underlying mechanism of this phenomenon using the TUNEL assay, and demonstrated that radiation-induced cell death of noninfected or Ax-mock-infected U251MG or D54MG cells was due to apoptosis, whereas we found that radiation-induced cell death of Ax-hp16-infected cells was not likely to be due to apoptosis. We also performed an electron microscopic assay, and detected apoptosis in noninfected U251MG cells, but not in Ax-hp16-infected U251 MG cells after irradiation (data not shown). The present results of PFGE and electron microscopic assay support our hypothesis that the cell death of irradiated Ax-hp16-infected cells was not due to apoptosis, suggesting that a different biochemical pathway or triggering mechanisms is involved in this mode of cell death.

Questions are raised by the present observations regarding the mechanism, whereby the irradiated Ax-hp16-infected cells exhibited cell-cycle arrest at the G1 phase (as indicated by flow cytometry) and the mechanism of cell death. The cellular roles of endogenous p16 are relatively poorly understood, but it is clear that its role is not limited to G1/S arrest. In HeLa cells, G2 delay correlates with elevated p16 levels, and loss of p16 expression results in loss of UVC-induced cell-cycle delay (Wang *et al*, 1996). Milligan *et al* (1998) reported that p16 acts specifically via its binding to cdk4 to produce G2 delay responses following UVC irradiation in melanoma cell lines. Moreover, p16 is found not only in the nucleus but also in the cytoplasm, especially in cancer cells (Sano *et al*, 1998; Chen *et al*, 1999), and plays an important role in the regulation of vascular endothelial growth factor (VEGF) expression and cell senescence (Harada *et al*, 1999).

It has been suggested that cancer cells can escape from G1/S arrest induced by p16, RB loss and Cyclin D1 amplification, via mechanisms that can over-ride the braking effect (Sherr *et al*, 1996). If there is a concomitant rapid exit from G2/S arrest induced by p16, there is no accumulation of tetraploid cells. In malignant cells, this exit can occur by abnormal nucleation, a term that includes not only nuclear budding/micronucleation and simple unequal nuclear division but also multipolar mitosis, repeated karyokinesis with acytokinesis and all divisions not associated with normal mitosis. The formation of these structures is not detectable by flow cytometry, because this method evaluates bare nuclei (Bhattathiri, 2001). Figure 2 indicates that the distribution of values around 2N and 4N is wider in the Ax-hp16-infected cells (both irradiated and nonirradiated), compared to the noninfected and Ax-mock-infected cells, a finding consistent with abnormal nucleation. Therefore, we used Giemsa staining to assay the induction of micronucleation, nuclear budding, binucleation and multinucleation individually in U251MG and D54MG cells 7 days after irradiation, with or without adenovirus infection (Figure 4).

In uninfected and Ax-mock-infected cells, there was an increase in cells with the above abnormalities, especially micro- and budded nucleation. Also, in Ax-hp16- and Ax-hp21-infected cells, there was an increase in cells with the above abnormalities, especially bi- and multinucleation. In unirradiated Ax-hp16- and Ax-hp21-infected U251MG cells, there was an increase in cells with the above abnormalities, especially bi- and multinucleation, but there was little change in unirradiated D54MG cells.

The situation is likely to be different when p16 and radiation are combined, with radiation inducing rapid proliferation and p16 facilitating rapid exit from G2/S arrest by abnormal nucleation. This abolishes apoptosis induced by G2/S arrest, yet promotes cell death by abnormal nucleation. The interaction of radiation and p16 in the morphological changes of abnormal nucleation has been reported. In skin cells, increased p16 expression with cytoplasmic predominance after UV irradiation correlates with the development of bi-, multi- and micronucleated cells (Milligan *et al*, 1998; Pavey *et al*, 1999). These observations suggest an unknown interaction between radiation and p16 in cytoplasm/membranes, which decreases cytokinesis and promotes abnormal nucleation.

Fueyo *et al* (1996) have shown that adenovirus-mediated p16 gene transfection alone significantly inhibits cell growth, a finding not obtained in the present study; Ax-hp16-infected cells used in the present study lost cell viability, as indicated in Figure 1D. This inhibition may be due to the induction of abnormal nucleated nonclonogenic cells. Obviously, the rates of survival and formation of multinucleated cells are likely to be higher in glioblastomas (Meyer-Puttlitz *et al*, 1997). Abnormal nucleation can also explain why apoptosis observed with radiation alone was absent when p16 transfection and radiation were combined. Obviously, p16 expression prevented radiation-induced apoptosis by promoting abnormal nucleation, thereby leading to cell death via this alternative mechanism. The situation can be viewed as radiation enhancing the effect of p16, resulting in an increase of radiation-induced cell death.

With p21, similar effects are possible, because p21 expression has also been found to be related to multinucleation (Okahashi *et al*, 2001). Mutinuclear osteoclast-like cell formation from osteoblasts is related to the upregulation of p21, with the majority of cells remaining at the G0/G1 phase (Tanaka *et al*, 2000). The integrity of p21 within centrosomes is related to apoptosis, multinucleation and normal mitotic progression (Li *et al*, 1999). It has been reported that, in MCF-7 cells, pacllitaxel induced the accumulation of p21 in cells with G2/M DNA content; this corresponds to cells in abnormal mitosis or an interphase-like state with multiple nuclei, the increase in p21 being subsequent to mitotic arrest and associated with exit from abnormal mitosis leading to the formation of cells with micronuclei (Barboule *et al*, 1997). It is possible that radiation also affects p21 localised in the centrosome complex, resulting in bi- or multinucleation.

Cell survival (assessed by clonogenic assay) markedly decreased in irradiated Ax-hp16-infected U251MG and D54MG cells, whereas cell viability (determined by trypan blue exclusion) only slightly decreased in the same cell lines; thus, the results of these two analyses are apparently not consistent. Physically dead cells are stained by trypan blue, but the formation of cellular fragmentation or cellular debris can occur over a period of several days, depending on the type of cell death; thus, trypan blue-positive (dead) cells are thought to reflect cells that died within a few days. On the other hand, abnormal nucleated cells are thought to be clonogenically dead, but not physically dead, and can synthesise DNA and continue to divide several times before death after irradiation (Bhattathiri, 2001). Unirradiated Ax-hp16-infected U251MG cells exhibited a modest increase in the proportion of abnormally nucleated cells, and 4-Gy irradiation of Ax-hp16-infected cells substantially enhanced this proportion (Figure 4A). After irradiation of Ax-hp16-infected D54MG cells, the frequency of abnormal nucleated cells increased (Figure 4B). Abnormal nucleated cells may not be completely detected by trypan blue analysis, but they are not clonogenic; thus, it is thought that the discrepancy was between cell viability and clonogenic assay.

A comparison between mock-infected and p16-infected D54MG cells (Figure 1E) shows that cell viability decreased identically for these cell populations until day 8 after irradiation. Teramoto *et al* (1995) examined whether E1-, E3-deleted adenovirus vectors affect the cell viability of human airway cells in primary culture, using trypan blue exclusion, and demonstrated that, as the MOI of adenovirus vectors is increased (to achieve effective gene transfer), the viability of infected cells is reduced concomitantly via apoptotic change. This reduced viability persisted for 5–7 days after vector administration. Tang *et al* (1997) demonstrated that irradiating cells at doses of 2–40 Gy prior to transduction could amplify recombinant adenoviral transgene products in a cell-type-specific manner. The apoptotic index of Ax-mock-infected D54MG cells increased, compared with uninfected cells, on days 3 and 7, with or without irradiation (Figure 3), but this trend was not exhibited by U251MG cells. One of the differences between U251MG and D54MG cells is p53 status; that is, p53 of U251MG is mutant, whereas that of D54MG is wild type. Therefore, it is possible that, in D54MG cells (but not in U251MG cells), irradiation amplified the recombinant adenoviral transgene products, activated a p53-dependent apoptotic pathway and increased apoptotic cell death, resulting in the reduction of cell viability, especially during the first 8 days after infection.

In the present study, continuous p16 gene expression had a significant cell killing effect after irradiation, but transient expression had little effect. Establishment of a promising strategy for gene therapy treatment of human malignant glioma patients using irradiation plus p16 gene expression requires continuous transgene expression. The adenoviral vectors used here are effective for gene transfer to many tissues, and confer high levels of expression of recombinant genes. However, the use of first-generation vectors usually results in transient transgene expression only, because of the development of a cellular immune response triggered by viral proteins expressed by adenovirus genes, the dose of virus administered, the promoter chosen to drive expression of the recombinant gene, innate immune mechanisms and direct cytotoxicity caused by the expression of viral genes (Morral *et al*, 1999). Future studies of prolongation of *in vivo* expression of adenovirally administered transgenes with minimised direct cytotoxicity may clarify the number of functions mediated by p16 and p21 after irradiation (eg, modulation of cytokinesis or karyokinesis) and contribute to gene therapy treatment of human malignant glioma patients.

To confirm whether the effect of Ax-hp16 infection plus irradiation was specific for human glioma cell lines, we also performed the clonogenic assay for other p16-null human cancer cell lines infected with Ax-hp16: the non-small-cell lung cancer cell line A549 (p21, undetectable protein expression; p53, wild type), and the gastric cancer cell line MKN45 (p21 and p53, wild type). The results indicate that the cell survival of these cancer cell lines was substantially decreased by Ax-hp16 infection (data not shown) (Kawabe *et al*, 2000; Yokozaki, 2000). These findings suggest that adenovirus-mediated p16 gene transfer increased radiosensitivity via nonapoptotic cell killing in these other human cancer cell lines.

The present results demonstrate that p16 gene transfer following radiation results in cell death. The modulation of the cell death response by intervention in a specific signal transduction pathway between radiation and p16 in cytoplasm/membranes, perturbing cytokinesis and promoting abnormal nucleation, may enhance the cytotoxic effect of radiation, thus improving the therapeutic ratio.
